# Closing the gap?—Trends in women’s full professorship at the medical faculty of the University of Rostock 1563–2018

**DOI:** 10.1186/s12909-026-09233-4

**Published:** 2026-04-22

**Authors:** Sabrina Bindrich, Nico Wille

**Affiliations:** 1 Rostock, Germany; 2 Department of Economics, Chair of Money and Credit, University of Rostock, Rostock, Germany

**Keywords:** Academia, Equity, Gender, Leadership, Participation

## Abstract

**Background:**

This study analyses the representation of female full professors at the Medical Faculty of the University of Rostock (UMR) between 1563 and 2018 based on the Catalogus Professorum Rostochiensium, while also examining the career paths and personal background of the professors.

**Methods:**

The data for this study was acquired from the Catalogus Professorum Rostochiensium (CPR), a biographical online lexicon, the university archive and the statistical department of the University of Rostock. Descriptive Statistics, the Mann–Whitney-U-Test and the Kruskal–Wallis-Test for independent samples followed by pairwise comparison with Bonferroni correction were used to analyse differences between subgroups like epochs and male or female professors.

**Results:**

The dataset includes 295 professors, 279 (94.57%) listed as male and 16 (5.42%) as female. There were no female full professors until 1964. While women are overrepresented in the lower ranks of academia, they remain underrepresented in the highest ranks: In 2018, 63.16% of students, 59.69% of the faculty’s graduates, 59.50% of newly graduated doctors and 33.33% of habilitations were women, while only 10.94% of professors at the UMR were female. In comparison, in 2018 female professors accounted for 23.69% of all professors in medicine in Germany. The first initiative for the promotion of women at the UMR mentioned in the archive files dates to 1962. The stomatological clinic was the first to create personalized development plans for women in academia and in total there were more female professors in orthodontics than male ones. Over the course of the study period, the proportion of female professors at the medical faculty increased noticeably, which is also reflected in a significant difference in appointment years between male and female professors (*p* < 0.001). In 1985, 9.76% of professors were women, while 55.90% of students were female. By 2024, these figures had risen to 18.31% of professors and 69.38% of students, respectively.

**Conclusion:**

Though significant progress has been made, further systematic changes are needed to achieve gender equity at the UMR. To make this possible, consequent initiatives by leaders, sufficient resources and continuous re-evaluation are needed.

## Background

Several international studies and meta-analyses have shown that women are still significantly underrepresented in leadership positions, such as full professorships, in Medicine [1–4]. This study aims to analyse the representation of female professors at the Medical Faculty of the University of Rostock between 1563 and 2018, while also focussing on their personal and educational background.

The University of Rostock was founded in 1419, making it the oldest university in the Baltic Sea region and the third oldest in Germany [5], and celebrated its 600th anniversary in 2019. To mark this occasion, the Catalogus Professorum Rostochiensium (CPR) was initiated with the purpose of compiling a comprehensive record of all professors in the university's history [6]. This provides us with a detailed data set to analyse how the average professor has changed throughout the years, while giving insight into the career paths of female academics particularly as well.

Women were only permitted to study medicine or other subjects at universities in Prussia starting from 1908 [7]. Even then, there was significant resistance to women's education within the academic community. The arguments largely came from the medical profession, particularly from neurologists and gynaecologists, and included points such as menstruation, reproduction, and a smaller brain size [8]. Based on her experience as the Women's Representative at FU Berlin, Färber [9] identifies three particular challenges for women's scientific careers in medicine: the medical and scientific career entails multiple burdens and a long qualification period. Additionally, there are exclusion mechanisms created by men that lead to the underrepresentation of women. Thirdly, societal prejudices against female physicians make it difficult for women to identify with a scientific career in medicine. According to Färber, the distinction between stereotypically male ("der Arzt" or the doctor) and typically female ("die Schwester" or the nurse) healthcare professions is strongly pronounced in Germany, among other things. [9]

At the University of Rostock, the earliest official Gender Equality Plan documented in the archival records of the medical faculty dates back to 1962 and provides an overview of female involvement at the faculty, while also outlining career options for women and measures to ease their everyday responsibilities [10]. At the time, representatives of various specialties raised concerns regarding women’s participation in research positions, citing potential conflicts with maternal responsibilities, issues surrounding maternity leave, and doubts about whether women’s performance in the field could equal that of men [11, 12]. Since then, initiatives promoting women and gender equality have continued to evolve, addressing persistent structural challenges and outlining measures aimed at increasing the representation of women in academia at the University of Rostock [13–17]. Since May 2021 there is also a mentoring program organized by the University of Rostock and the medical faculty for female physicians who are aiming for a leadership position in the clinic or a professorship. The program assists the participants in planning their scientific career through one-on-one mentoring [18].

Given the additional struggles mentioned for women in comparison with their male counterparts, it is to be expected, that there are not only fewer female full professors at medical faculties in Germany, but that the age at appointment is also higher for them, especially, when their scientific career was interrupted by pregnancies and childcare. The analysis of the German University Professors Directory shows that up to 1994, 328 habilitated women were listed in the medical fields [9]. Of these, 120 were private lecturers, 76 were non-tenured professors, 12 were honorary professors, 21 women held full professorships (C 4), and 93 were university professors (C 3) [9]. Only 6.4 percent of habilitated women were currently full professors and, therefore, also department heads [9]. Carnes and Bairey Merz also note that stereotypical assumptions about what a leader should look like overlap much more strongly with male stereotypes than with female stereotypes [19].

Research shows that in business, organizations with more diversity have a better economic performance [20]. A diverse team exhibits better problem-solving skills and more diverse executive boards lead to improved profitability and business success in general [21]. In medicine providers that show more similarities with their patients result in better communication, decision-making and adherence to treatment plans [22]. Moreover a diverse training environment appears to improve learning outcomes in higher education [23] and provides a better preparation for working effectively in diverse environments [24]. In their report on female leadership in a surgical department, Rakestraw et al. come to the conclusion that by promoting qualified female candidates to leadership positions, one can provide role models for upcoming female surgeons [25]. On the other hand, Rosenkranz et al. [26] point out that a majority of leadership positions are still held by white men. Therefore, it is essential to involve them in solving the problem to enable career advancement opportunities for underrepresented groups [26]. Their appeal is that white men must take an active role as mentors, sponsors, and allies in the fight for greater diversity [26].

### Hypotheses

Based on the studies mentioned above the authors come to the following hypotheses:

1. The proportion of women among full professors at the medical faculty remains significantly lower than that of male professors until today.
2. The proportion of female professors is significantly below the proportion of female graduates at the medical faculty of the University of Rostock.
3. Women are likely to be significantly older than men when appointed as professors.

## Methods

The data for this study was acquired from the Catalogus Professorum Rostochiensium (CPR), a biographical online lexicon with the goal to document every professor at the University of Rostock since its foundation in 1419 [6]. From 1563 to 2018 the catalogue holds a complete record of all professors during that time span, before 1563 and after 2018 the data is incomplete [6].

The examined parameters are the number of male and female professors, duration of professorship, age at appointment as professor, time span between doctorate and appointment, number of appointments per epoch, professorships after their time at the University of Rostock and family background. Because certain variables, including the time span between the doctorate and appointment, age at appointment and duration of professorship, are not explicitly listed in the catalogue, these values were calculated by the authors of this study on the basis of the years provided. This study focusses on the Faculty of Medicine as one of the university’s founding faculties [27].

Additional statistical data, namely number of students, number of graduates, number of doctorates, number of professors including junior professors and the proportion of women in the above categories, and data on initiatives for the promotion of women was acquired from the university archive and the statistical department of the University of Rostock.

The CPR divides the study period into seven historical epochs: 1563–1827 from the Formula Concordiae till the end of the Co-patronage of the sovereign and the city; 1760–1789 the University of Bützow; 1827–1918 from the end of the Co-patronage till the end of World War One; 1918–1933 the Weimar Republic; 1933–1945 the Third Reich; 1945–1990 post-war and East German period; 1990–2018 contemporary period [28]. These time periods will be considered individually in the study and comparisons between them will be made. Since the epochs in the catalogue overlap, the following timespans were used in our analysis: 1 January 1563–31 December 1826, 1 January 1760–31 December 1789 (Bützow), 1 January 1827–31 December 1917, 1 January 1918–31 December 1932, 1 January 1933–31 December 1944, 1 January 1945–31 December 1989, 1 January 1990–31 December 2018. For detailed information on the historical context please refer to the appendix.

In the catalogue, professors are categorized as either male or female. We retained this classification without independently reassessing gender attribution. In this paper, the terms *female* and *woman* are used interchangeably in accordance with the catalogue’s categorization.

### Study collective

This study includes all full professors of the Faculty of Medicine listed in the Catalogus Professorum Rostochiensium (CPR) between 1 January 1563 and 31 December 2018. In the catalogue several professorship types are considered a full professorship. In this study we focus on the following types: full professorship (" ordentliche Professur "), unspecified professorship, ducal professorship ("herzoglich"), municipal professorship ("rätlich"), C4 professorship (highest level of professorship according to the *C-Besoldung (C1-C4)* salary system till 31 December 2004 [29]) and W3 professorship (highest level of professorship according to the current *W-Besoldung (W1-W3)* salary system).

### Statistical analysis

The statistical analysis was conducted using IBM SPSS Statistics, version 29. The level of statistical significance was set at 5% (α-error = 0.05). Normality of continuous variables was assessed using the Shapiro–Wilk test. Although a small number of variables showed approximate normal distribution, the majority did not. In addition, the small number of female professors (*n* = 16) limits the reliability of parametric tests. Therefore, non-parametric tests were applied consistently across all analyses. The Mann–Whitney-U-Test and the Kruskal–Wallis-Test for independent samples followed by pairwise comparison with Bonferroni correction were used to analyse differences between subgroups like epochs and male or female professors. The continuous and categorical variables such as mean, count, standard deviation and percentage, were calculated with Microsoft Excel 2019, version 1808. Descriptive statistics were calculated using all available data for each variable; therefore, sample sizes may vary due to missing values. The same approach was applied to the non-parametric tests, which were conducted using all available cases for the variables included in each analysis.

## Results

The dataset includes 295 professors that met the inclusion criteria, 279 (94.57%) were listed as male and 16 (5.42%) as female. The data was 100% complete for the variable sex. The Mann–Whitney-U-Test for independent samples shows no significant differences for age at appointment as a professor (*p* = 0.432), duration of the professorship (*p* = 0.356) and time between doctorate and professorship between the male and the female subgroup. The proportion of female professors differed significantly over the study period (*p* < 0.001) with a significant increase in later epochs. The first female professor at the medical faculty of the University of Rostock was appointed as a professor for internal medicine in 1964 at the age of 45 years.

The year of birth was given for 96.61% of the professors with 1530 as the earliest and 1978 as the latest within the dataset. For the female professors the date of birth was listed in 87.50% with a minimum of 1919 and a maximum of 1974. The data for the male professors was 97.13% complete with a minimum of 1530 and a maximum of 1978. The place of birth was listed in 92.88% (81.25% for female professors; 93.55% for male professors) of cases and shows a clustering within Rostock and the rest of Mecklenburg-Western-Pomerania (together 19.34%). It also shows that 19.34% were born outside of Germany based on today’s borders. It is important to acknowledge that many of these territories used to be part of Germany and would not have been considered foreign at the time. The Kruskal–Wallis-Test for independent samples shows no significant differences between the different birth places for the categories age at appointment (*p* = 0.734), duration of professorship (*p* = 0.111) and time span between doctorate and professorship (*p* = 0.207).

The data on the religious beliefs of the professors was 49.15% (56.25% for female professors; 48.75% for male professors) complete and reveals that the majority was evangelical (Table 1). The Kruskal–Wallis-Test for independent samples shows no significant differences based on religious beliefs for the categories age at appointment (*p* = 0.145) and time span between doctorate and professorship (*p* = 0.086). There was a difference for the category duration of professorship (*p* = 0.023) but the pairwise comparison with Bonferroni correction showed no significant results for the different religions.

**Table 1:** Overall distribution of the professors’ religion according to the CPR

Religion	Total number	Male professors	Female professors
Evangelical	83 (57.24%)	77 (56.62%)	6 (66.67%)
Evangelical Lutheran	27 (18.62%)	27 (19.85%)	0 (0.00%)
Roman Catholic	16 (11.03%)	15 (11.03%)	1 (11.11%)
None	10 (6.90%)	9 (6.62%)	1 (11.11%)
Catholic	6 (4.14%)	5 (3.68%)	1 (11.11%)
Protestant reformed	2 (1.38%)	2 (1.47%)	0 (0.00%)
Jewish	1 (0.69%)	1 (0.74%)	0 (0.00%)

Totals may not sum to exactly 100% due to rounding

Year of death was given in 62.71% with 1589 being the earliest and 2025 the latest within the dataset. It should be noted that a proportion of the missing data (37.29%) corresponds to professors who were still alive at the time of data collection. For female professors the data was 37.50% complete with a minimum of 2000 and a maximum of 2023. For the male professors the year of death was given in 64.16% with a minimum of 1589 and a maximum of 2025.

The profession of the professor’s father was listed in 62.71% cases and shows that 13.40% were professors themselves (Table 2). The mother’s profession was given in 18.64% cases and reveals that while 12.73% were physicians, none was a professor herself. Almost one-third of them were homemakers. For the female professor’s data on the father’s profession was available in 43.75% of cases and on the mother’s profession in 12.50% of cases. The data on the father’s profession was 72.40% complete for the male professors and the data on the mother’s profession 19.00%. The Kruskal–Wallis-Test for independent samples showed no significant differences between the different professions of both father and mother for the categories age at appointment (father *p* = 0.573, mother *p* = 0.935), duration of professorship (father *p* = 0.578, mother *p* = 0.540) and time span between doctorate and professorship (father *p* = 0.802, mother *p* = 0.778).

**Table 2:** Overall distribution of the profession of the professors’ fathers and mothers

Profession	Father total	Mother total	Father male professors	Mother male professors	Father female professors	Mother female professors
Clergyman/Academic	47 (22.49%)	8 (14.55%)	46 (22.77%)	8 (15.09%)	1 (14.29%)	0 (0.00%)
Apprenticeship	37 (17.70%)	11 (20.00%)	35 (17.33%)	11 (20.75%)	2 (28.58%)	0 (0.00%)
Physician	30 (14.35%)	7 (12.73%)	30 (14.85%)	7 (13.21%)	0 (0.00%)	0 (0.00%)
Professor	28 (13.40%)	0 (0.00%)	27 (13.37%)	0 (0.00%)	1 (14.29%)	0 (0.00%)
Leadership position	22 (10.53%)	1 (1.82%)	19 (9.41%)	0 (0.00%)	3 (42.87%)	1 (50.00%)
Manual work	21 (10.05%)	7 (12.73%)	21 (10.40%)	7 (13.21%)	0 (0.00%)	0 (0.00%)
Self-employed/owner	17 (8.13%)	1 (1.82%)	17 (8.42%)	1 (1.89%)	0 (0.00%)	0 (0.00%)
Doctor (other than med.)	6 (2.87%)	1 (1.82%)	6 (2.97%)	1 (1.89%)	0 (0.00%)	0 (0.00%)
Artist	1 (0.48%)	2 (3.64%)	1 (0.50%)	2 (3.77%)	0 (0.00%)	0 (0.00%)
Homemaker	0 (0.00%)	17 (30.91%)	0 (0.00%)	16 (30.19%)	0 (0.00%)	1 (50.00%)

Totals may not sum to exactly 100% due to rounding

The data on the epoch in the catalogue was 100% complete and shows an increase in appointments in recent years with a maximum in the last epoch (Table 3).

**Table 3:** Total number of professor appointments by the epochs in the CPR

Epoch	Number	Male professors	Female professors
1563–1826	34 (11.53%)	34 (12.19%)	0 (0.00%)
1760–1789 (Bützow)	1 (0.34%)	1 (0.36%)	0 (0.00%)
1827–1917	44 (14.92%)	44 (15.77%)	0 (0.00%)
1918–1932	17 (5.76%)	17 (6.09%)	0 (0.00%)
1933–1944	14 (4.75%)	14 (5.02%)	0 (0.00%)
1945–1989	91 (30.85%)	85 (30.47%)	6 (37.50%)
1990–2018	94 (31.86%)	84 (30.11%)	10 (62.50%)

Totals may not sum to exactly 100% due to rounding

The Kruskal–Wallis-Test for independent samples indicates significant differences for the categories age at appointment (*p* < 0.001), duration of professorship (*p* = 0.022) and timespan between doctorate and professorship (*p* < 0.001) in between the epochs (Table 4). The following pairwise comparison with Bonferroni correction revealed that the age at appointment was significantly lower in the first epoch (1563–1826) than in 1933–1944 (*p* = 0.026), 1945–1989 (*p* < 0.001) and 1990–2018 (*p* < 0.001). In epoch three (1827–1917) it was also significantly lower than in 1945–1989 (*p* < 0.001) and 1990–2018 (*p* < 0.001). Moreover, in epoch one the duration of professorship was significantly longer than in 1990–2018 (*p* = 0.012). The time span between doctorate and professorship was significantly shorter in 1563–1826 than in 1918–1932 (*p* = 0.039), 1933–1944 (*p* = 0.039), 1945–1989 (*p* < 0.001) and 1990–2018 (*p* < 0.001). In 1827–1917 it was also shorter than in 1945–1989 (*p* < 0.001) and 1990–2018 (*p* = 0.032).

**Table 4:** Overall comparison of different variables between the epochs in the CPR

Epoch	Place of birth	Father’s profession	Mother’s profession	Age at appointment	Duration of professorship	Years between doctorate and professorship	Professorship afterwards
1	Rostock: 29.41% MV: 14.71%	Professor: 13.79% Physician: 24.14%	Professor: 0.00% Physician: 0.00%	Median 34.50; Mean 34.76 (SD: 8.503) ; min 24; max 56	Median 19.00; Mean 21.82 (SD: 13.183); min 2; max 53	Median 6.00; Mean 8.13 (SD: 8.597); min −6; max 30	8.70%
2	Rostock: 100% MV: 0	Professor: 0.00% Physician: 0.00%	Professor: 0.00% Physician: 0.00%	34 (*n* = 1)	20 (*n* = 1)	10 (*n* = 1)	100.00%
3	Rostock: 2.27% MV: 6.82%	Professor: 20.51% Physician: 23.08%	Professor: 0.00% Physician: 0.00%	Median 37.00; Mean 38.07 (SD: 7.108) ; min 27; max 56	Median 12.00; Mean 14.74 (SD: 10.644); min 1; max 45	Median 13.00; Mean 13.66 (SD: 6.513); min 3; max 31	37.50%
4	Rostock: 0.00% MV: 0.00%	Professor: 12.50% Physician: 6.25%	Professor: 0.00% Physician: 0.00%	Median 43.00; Mean 42.53 (SD: 4.094); min 36; max 49	Median 10.00; Mean 12.53 (SD: 9.520); min 1; max 34	Median 15.00; Mean 15.88 (SD: 5.122); min 4; max 23	58.82%
5	Rostock: 7.14% MV: 7.14%	Professor: 7.14% Physician: 21.43%	Professor: 0.00% Physician: 0.00%	Median 43.50; Mean 44.29 (SD: 5.823)); min 34; max 58	Median 12.00; Mean 15.50 (SD: 12.030); min 3; max 51	Median 14.50; Mean 17.00 (SD: 6.409); min 8; max 32	35.71%
6	Rostock: 7.06% MV: 15.29%	Professor: 4.11% Physician: 9.59%	Professor: 0.00% Physician: 0.00%	Median 46.00; Mean 47.02 (SD: 7.571); min 33; max 69	Median 15.00; Mean 14.58 (SD: 7.230); min 1; max 31	Median 19.00; Mean 20.27 (SD: 7.956); min 6; max 44	12.94%
7	Rostock: 6.33% MV: 10.13%	Professor: 18.92% Physician: 16.22%	Professor: 0.00% Physician: 29.17%	Median 46.00; Mean 46.38 (SD: 5.782); min 35; max 64	Median 11.00; Mean 12.22 (SD: 6.988); min 2; max 26	Median 18.00; Mean 18.22 (SD: 6.658); min 7; max 36	6.52%

MV refers to Mecklenburg-Vorpommern

The median age at appointment has increased from 34.50 in the first epoch (1563–1826) to 46.00 years in the sixth epoch (1945–1989) and stayed at that level the following epoch (Table 4). In the same time span, the median amount of years between doctorate and professorship has increased from 6.00 to 19.00 before decreasing again in the last epoch. The median duration of professorship fluctuated between the epochs but was the highest in the first epoch (19.00 years) and the lowest in the fourth (10.00 years), followed by the final epoch (11.00 years).

The type of professorship was given 100% and reveals that almost half of the professors held full professorships without further classification (Table 5). According to the Kruskal–Wallis-Test for independent samples there is a significant difference between the different types of professorships in the categories age at appointment (*p* < 0.001), duration of professorship (*p* = 0.021) and time span between doctorate and professorship (*p* < 0.001). The following pairwise comparison with Bonferroni correction indicates that ducal professors were significantly younger when they were appointed than full professors (*p* < 0.001), professors without further specification (*p* < 0.001), C4 professors *(p* < 0.001) and W3 professors (*p* = 0.004). C4 professors were also significantly older than full professors (*p* < 0.001) and municipal professors (*p* = 0.007) at appointment. Moreover, ducal professors appeared to have been significantly longer in office than professors without further specification (*p* = 0.027). They also had a shorter time span between doctorate and professorship than full professors (*p* < 0.001), professors without further specification (*p* < 0.001), C4 professors (*p* < 0.001) and W3 professors (*p* = 0.040). For C4 professors there were significantly more years between doctorate and professorship than for full professors (*p* = 0.008) and municipal professors (*p* = 0.011).

**Table 5:** Overall distribution of type of professorship in the CPR

Professorship	Total number	Male professors	Female professors
Full	141 (48.12%)	136 (49.10%)	5 (31.25%)
unspecified	61 (20.81%)	53 (19.13%)	8 (50.00%)
Council	14 (4.78%)	14 (5.05%)	0 (0.00%)
Ducal	19 (6.48%)	19 (6.86%)	0 (0.00%)
C4	43 (14.68%)	40 (14.44%)	3 (18.75%)
W3	15 (5.12%)	15 (5.42%)	0 (0.00%)

Totals may not sum to exactly 100% due to rounding

The data on age at appointment was 96.61% complete and was 43.54 (± 8.18) years on average with a median of 44.00 years, a minimum of 24 years and a maximum of 69 years. For the female professors the data was 87.50% complete with a mean of 44.86 (± 6.06) years, a median of 46.00 years, a minimum of 35 years and a maximum of 56 years. For the male professor’s data on the age at appointment was 97.13% complete and showed an average of 43.47 (± 8,28) years with a median of 44.00 years, a minimum of 24 years and a maximum of 69 years.

The duration of professorship at the University of Rostock could be calculated in 81.69% of cases. For professors who were still in office at the time of the data collection it was not possible to calculate the total duration of their professorship. The mean time was 15.16 (± 9.69) years with a median of 14.00 years, a minimum of 1 and a maximum of 53 years. For female professors the time span could be calculated for 62.50% and showed an average of 12.10 (± 7.23) years, a median of 12.00 years, a minimum of 3 years and a maximum of 23 years. For the male professors it could be calculated for 82.80% with a mean of 15.29 (± 9.77) years, a median of 14.00 years, a minimum of 1 year and a maximum of 53 years.

Data was 100% complete for the beginning of the professorship, 81.69% (62.50% for the female professors; 82.80% for the male professors) complete for the end of the professorship and 91.86% (81.25% for the female professors; 92.47% for the male professors) complete for the year of obtaining a medical doctorate. The mean time between doctorate and professorship was 16.73 (± 8.11) years with a median of 16.00 years, a minimum of −6 years (the professor held a Magister Artium before acquiring a medical doctorate during his professorship resulting in a special case) and a maximum of 44 years. For the female professors the mean time was 18.00 (± 6.26) years with a median of 18.00 years, a minimum of 7 years and a maximum of 28 years. The mean time for the male professors was 16.66 (± 8.20) years with a median of 16.00 years.

In 82.71% the institute at the medical faculty was given. The Kruskal–Wallis-Test for independent samples showed no differences between the different institutes for the category duration of professorship (*p* = 0.481). For the categories age at appointment (*p* = 0.023) and time span between doctorate and professorship (*p* = 0.026) there were significant differences between institutes that did not show in the following pairwise comparison with Bonferroni correction. The female professors were employed by the following institutes (Table 6) and data was 100% complete. For the male professors, the data on the institute was 81.72% complete.

**Table 6:** Distribution of institutes by presence of female professors in the CPR

Institute	Female professors	Male professors
psychiatry	3 (18.75%)	13 (5.70%)
orthodontics	3 (18.75%)	1 (0.44%)
medical microbiology	2 (12.50%)	5 (2.19%)
internal medicine	2 (12.50%)	26 (11.40%)
medical biochemistry	1 (6.25%)	6 (2.63%)
anaesthesiology & intensive care	1 (6.25%)	2 (0.88%)
paediatric	1 (6.25%)	16 (7.02%)
dental clinic	1 (6.25%)	9 (3.95%)
biomedical technology	1 (6.25%)	2 (0.88%)
experimental surgery	1 (6.25%)	0 (0.00%)

In 92.20% there was information on the career of the professors after they left the University of Rostock in the catalogue. 18.01% became professors at other universities either in Germany or abroad. For the female professors the date was 93.75% complete and only one of them (6.25%) became a professor at another university afterwards. The data for the male professors was 92.11% complete and 48 (18.68%) continued as professors at other universities. The Mann–Whitney-U-Test for independent samples followed by a pairwise comparison shows that professors who did not continue as professors at other universities were significantly older when they were appointed (*p* < 0.001) and their professorship at the University of Rostock lasted significantly longer (*p* < 0.001) than it was the case for professors who joined a different university as professors. The time between doctorate and professorship was significantly (*p* < 0.001) shorter in the subgroup of professors who continued as professors at another university.

### University of Rostock higher education statistics

**Fig. 1:**
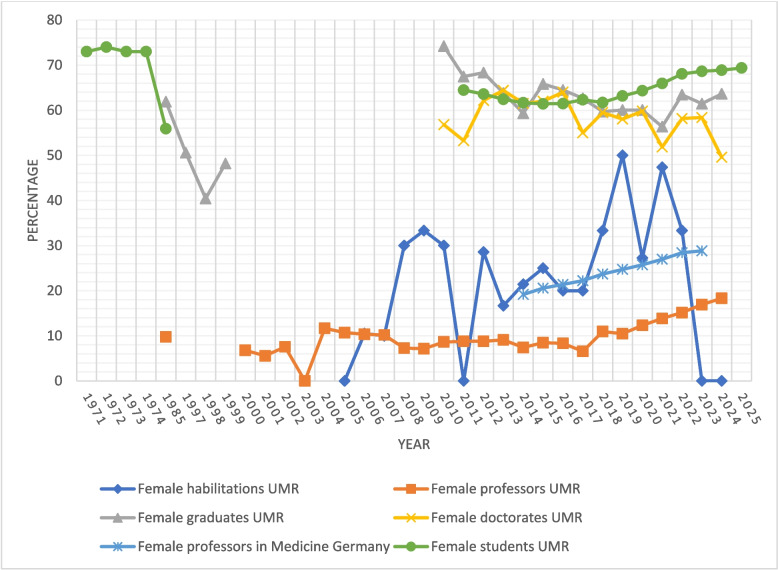
Development of female representation in medical academia. Notes: For graduates and doctorates the years 2010—2017 only include the degree programs in Human Medicine, Dentistry, and Medical Biotechnology. For the students the same applies for the years 2011–2018. For the habilitations and professors up to and including 2017, the figures refer exclusively to positions financed from the University of Rostock's budgetary funds; from 2018 onwards, third-party and special funds are also included in the calculation. However, these constitute only a very small proportion. Data from the internal University Statistics is not available to the public. The full tables (Tables 7, 8, 9 and 10) are in the Appendix. Sources: [16, 17, 30], University Statistics [31] and University of Rostock archive data [16, 17, 32–34]

For data protection reasons, datasets with groups of fewer than five individuals in a category may only be used for internal university purposes. Therefore, the affected data are provided only as percentages. Moreover, the year indicated varies depending on the data source. Because the data in this chapter were compiled from a variety of sources, including archival records, temporal references are not fully consistent across all tables and figures. To improve readability and comparability, years are reported as full calendar years throughout. When only a single year is given, it refers to both the winter semester (WS) and the summer semester (SS) with final examinations in that year; for example, 2024 includes WS 2023/24 and SS 2024. In some sources, only a general year was specified that encompassed both semesters, whereas in others only the winter semester was indicated. In the latter case, the year at the end of the winter semester was used for consistency; thus, the winter semester 2020/21 is recorded as 2021. Figure 1 displays the development of female representation in medical academia over time.

The proportion of female graduates was over 60% in 1985 but decreased in subsequent years, falling significantly below 50% by 1998. In recent years it fluctuates but has consistently remained above 50% since at least 2010 (Fig. 1). Similarly, since 2010 the percentage of female doctoral graduates has been above 50%, except in 2024, although a downward trend is observable.

Over the past 20 years, the proportion of women completing their habilitation at the medical faculty has fluctuated but generally ranged between one-quarter and one-third. However, there have been years without habilitations completed by women. The *Habilitation* formally recognizes a scholar’s ability to conduct independent research and teaching in a specific academic field. It requires a doctoral degree and is based on a scholarly dissertation, a trial lecture, and an academic colloquium. Successful completion grants the *facultas docendi* and the *venia legendi*, authorizing the holder to independently teach at the university according to faculty regulations [35]. In contrast, the proportion of women who completed their habilitation was around 50% in 2019 and 2021. Regarding professorships held by women, there is an overall upward trend, yet the percentage has never exceeded 20%, with its peak at 18.31% in 2024 (Fig. 1).

When looking at Germany as a whole, it is evident that there is an upward trend in the proportion of female professors. However, in 2023, less than one-third of these positions were held by women (Fig. 1).

The proportion of female students was highest in the 1970 s, with nearly three-quarters, but it has never fallen below 50% since then. Overall, there has been an upward trend in recent years, with female students currently making up about two-thirds of the medical faculty's student body (Fig. 1).

## Discussion

As hypothesized, there were no female full professors till 1964, and women present a minority with only 5.42% of all full professors at the medical faculty in the catalogue being female. This remains visible when only considering the most recent epoch from 1990 to 2018, supporting our hypothesis that women are still a minority among full professors at the UMR today. During that time ten out of 94 professors were female resulting in a proportion of 10.64%. This is consistent with data from the university’s statistics department, which shows that while in 2018, 33.33% of habilitations at the University Medicine Rostock were achieved by women only 10.94% of professors at the UMR were female. In comparison with the rest of Germany data shows that in 2018 female professors made up 23.69% of all professors in Medicine in Germany [30], which means that the German average is twice as high as the women’s proportion at the University of Rostock.

The increase in the number of female professors should also be viewed in the broader context of women’s social and political emancipation. In many European countries, women gained the right to vote only in the early twentieth century, for example in Germany in 1918 [36]. Economic autonomy also emerged relatively late: in Germany full financial autonomy was introduced in 1977 [37]. Despite these advances, women remain underrepresented in senior academic positions worldwide, with female professors accounting for roughly one quarter to one third of professorships in the European Union [38].

When looking at the student numbers the contrast becomes even starker, underlining our hypothesis that the proportion of female professors is far below the proportion of female graduates at the medical faculty of the University of Rostock. In the winter semester of 2018/19, 63.16% of students were female. The same year 59.69% of the UMR’s graduates and 59.50% of newly graduated doctors were women. Like our data, international studies highlight as well that while women are overrepresented in the lower ranks of academic careers [3, 4] they are less likely to reach the senior ranks of academic medicine [2–4, 39, 40]. An Austrian study on female participation in medicine shows that in 2019/20, 53.8% of medical graduates in Austria were women, while only 30% of all professor positions at the three Austrian state Medical Universities were held by women [41].

A report by the European Commission with data from 2021 shows that significant gender disparities persist in academic career progression within the European Union [38]. Although women represented 48% of doctoral graduates, indicating that gender balance at this stage has largely been achieved, they remain substantially underrepresented in senior academic positions. Women hold only 30% of top academic positions (Grade A) across the EU, even in disciplines where they are generally well represented. For example, women account for only 34% of Grade A staff in Humanities, Arts, and Social Sciences and 33% in Medical and Health Sciences. At the doctoral level, gender balance, defined as women representing between 40 and 60% of graduates, has been reached in almost all EU Member States, with the exception of Cyprus, where women constitute a clear majority (66%), and Austria, where women remain slightly underrepresented at just under 40%. In the field of Health and Welfare, women account for 59.5% of doctoral graduates, making it one of the most female-dominated areas of doctoral education. Moreover, at the EU level, the largest share of women doctoral graduates complete their degrees in Health and Welfare (28%), whereas among men, the highest proportion graduate in Natural Sciences, Mathematics and Statistics (26%). Together, these figures highlight the persistent “leaky pipeline” in academia: despite achieving gender balance, or even a majority, at the doctoral level in some fields, women continue to be significantly underrepresented in the highest academic ranks. [38]

A report by the British Medical Association points out that the underrepresentation of women is also visible in expert groups, advisory or policy committees and peer-review panels [42]. Moreover, a study from 2013 on citation and publication patterns demonstrates that female authors are cited systematically less than their male counterparts [43]. This is attributed to the fact that women tend to self-citate less and men are more likely to cite papers authored by other men [43]. That pattern is especially relevant to our discussion because the quality of publications and number of citations plays a major role in the application process for professors at the University of Rostock [44, 45] and other German universities [46].

At the medical faculty of the University of Rostock there were no initiatives for the promotion of women mentioned in the archives before 1962 and the first drafts mainly focussed on the relief of everyday responsibilities for female professionals [10]. During that time serious doubts were voiced on women in academic careers at the faculty [12] and some even questioned the benefit of women in academic careers in general [11]. Today, women continue to face discrimination based on their sex and sexual harassment especially in the academic field as several studies point out [2, 47–49]. This debate may be part of the reason why there were no female full professors until 1964 and their proportion continued to be low after that.

In 2008 the Women Professors Program was initiated in Germany with the goal of achieving structural changes to improve gender equality at higher education institutions [50]. In her article Löther assesses the effect of this program by showing that the proportion of female professors at participating institutions increased more than that at non-participating institutions [50]. Her analysis confirmed that the participation in the program had an independent effect on the proportional increase of female professors [50]. However, she also points out that said increase was more pronounced in western German states and Berlin than in eastern German states, without going into detail regarding the causes [50]. Löther attributes the increase to two main factors: the implementation of a gender action plan and the professorships supported by the start-up financing provided through the program [50]. While King et al. criticize that recent family-friendly approaches at the workplace often focus on the women and are rarely complemented by measures supporting male caring [51], the initiatives for the promotion of women at the University of Rostock also include family-friendly initiatives that are gender-unspecific [14].

In the following fields there were more female professors than male professors: orthodontics and experimental surgery. Interestingly, while there were female professors in internal medicine which comprises the specialty with the most professorships in total, there were no female professors in surgery as another major specialty which accounts for 8.77% (22) of the male professorships. International studies on women in leadership positions in surgical specialties show a severe lack of women across subspecialties in chairmanship positions and full professorships [1], supporting our observations. The most frequent specialties among female professors in the catalogue were psychiatry and orthodontics. This may have to do with the fact that the Stomatological Clinic was the first clinic in the archives that aimed to create personalized development plans for women intended to acquire the Facultas Docendi [52], which leads to the conclusion that the culture at that clinic may have been more welcoming towards women than other departments.

Lightfoote et al. call upon all medical societies to lead a cultural shift by emphasizing the importance of inclusion and diversity to improve their clinical quality [53]. To facilitate this, Westring et al. propose “an integrated framework for gender equity in academic medicine” [54]. They see four main factors needed for gender equity: equal access to resources and opportunities, reducing unconscious gender bias, enhancing work-life balance and the engagement of leadership. Concrete measurements include a good mentor–mentee fit, coaching on unconscious bias and providing sufficient resources for career development. An essential factor is the involvement of leadership on every level; the institution’s culture must be addressed from within and from the outside for example by funding agencies. The authors suggest a step-by-step plan: in the beginning the current status regarding gender equity within an institution needs to be assessed by internal and external leadership; afterwards financial resources and personnel must be provided to implement evidence-based solutions; and finally, the progress must be evaluated in a transparent manner. It is also emphasized that developmental programs aimed at the women themselves shift the responsibility from leadership to those women. The framework goes even further and addresses other important institutions for an academic career like journals, honorary societies and committees on medical education, to examine their operations for gender bias [54]. Similar suggestions are made by Acosta et al. in order to achieve gender equity [48]. While many parts overlap, like the examination of biases and the involvement of leadership, they also emphasize the importance of inclusion and diversity making an intersectional approach essential [48]. For institutional change both top-down and bottom-up approach or a combination of both are proposed [54, 55].

Beyond the conceptual frameworks discussed above, gender equality in academia is also shaped by broader policy initiatives and funding structures. At the European level, the European Union has introduced several strategies aimed at promoting gender equality across society, including research and higher education [56]. The EU Gender Equality Strategy 2020–2025 defined key objectives such as closing gender gaps in the labour market, increasing women’s participation in decision-making positions, and challenging gender stereotypes [56]. Building on these efforts, the Gender Equality Strategy 2026–2030 further emphasizes gender equality across all areas of life, including education, health, and leadership, and introduces initiatives such as an Action Plan on Women in Research, Innovation and Start-ups as well as the “Girls Go STEM” initiative [56]. In addition, EU research funding programmes increasingly incorporate gender equality requirements. Within the Horizon Europe framework programme, many public research organisations and universities must have a Gender Equality Plan (GEP) in place in order to be eligible for funding [57]. Recent policy initiatives, including the Roadmap for Women’s Rights (2025) and the Report on Gender Equality in the EU (2025), highlight that although some progress has been made, particularly regarding women’s leadership, structural inequalities persist and continued policy action remains necessary [56].

Contrary to our hypothesis that women were likely to be older than men when appointed as professors, there were no significant differences for age at appointment as a professor (*p* = 0.432), duration of the professorship (*p* = 0.356) and time between doctorate and professorship between the male and the female professors in this study. In contrast a UK report from 2012 shows that men were more likely to reach senior grades in their academic careers at a younger age than women [40]. A study from the U.S. also found that men were promoted to associate and full professors more quickly and more often than women, while women were appointed as assistant professors earlier [4].

The median age at appointment increased significantly (*p* < 0.001) during the study period, rising from 34.50 years in the first epoch (1563–1826) to 46.00 years in the final epoch (1990–2018). The age of appointment in the first epoch is in the same range as statistics from other European universities of that time suggest [58–60]. For today, one study states a mean age of 43 for full professors at appointment without differencing between faculties [61]. The study emphasizes that new types of professorship like tenured professors or junior professors were introduced to lower the mean age of appointment in Germany but in medicine the traditional habilitation system remains common [61]. A recent study also suggests that PhD graduates over the age of 40 years are subject to age discrimination in a way that benefits their path to professorships at universities of applied sciences [62]. In line with the median age at appointment, the median amount of years between doctorate and professorship has significantly (*p* < 0.001) increased from 6.00 to 18.00 years. The markedly increased life expectancy in Germany [63] may help to explain the higher age at appointment and, accordingly, the longer interval between doctoral degree and professorship. In earlier eras, considerably less lifetime was available to pursue these career steps, whereas today the time available and necessary for academic qualification and advancement is substantially longer. The median duration of professorship fluctuated between the epochs but was the highest in the first epoch (19.00 years) and visibly lower in the final epoch (11.00 years). This demonstrates a significant decrease over the study period in total (*p* = 0.012) and is in line with the professorship types: ducal professors were significantly younger than most other types of professors at appointment while C4 professors were significantly older than full professors which make up a lot of the professorships of the earlier epochs and municipal professors. One factor contributing to the shorter duration of professorships may be the mandatory retirement ages stipulated by state civil service laws [64, 65]. Whereas professors historically often remained in office until their death, they are now formally retired upon reaching these age limits. In most federal states, professors currently retire at the end of the semester in which they reach the statutory retirement age of 67 [66].

In total 27.75% of the professors in the study collective had fathers that were either physicians, professors or both and 12.73% had mothers that were physicians themselves. The percentages fluctuate over the different epochs and are not significantly higher in the first epoch than in the last. This stands in contrast to a study by La Croix and Goni that examines scholars lineages in preindustrial Europe and observes a decrease in nepotism over the study period [67]. While having family members in the same professional area does not necessarily lead to nepotism it is likely to serve as an advantage. A recent study suggest that a higher socioeconomic status of the parents and parents with PhDs in particular lead to a disproportionate access to professorships, this being a stable observation over the last 50 years [68].

### Limitations

Given the retrospective nature of the Catalogus Professorum Rostochiensium no causal conclusions can be derived from the data, making this a descriptive study only. Another major limitation of this study is the incompleteness of the data. This issue is particularly pronounced in the most recent epoch, where stricter data privacy regulations have restricted access to personal information. Consequently, some individuals may be underrepresented or missing entirely from the dataset, introducing potential bias in the analysis and interpretation of trends over time. The small sample size of female professors (*n* = 16) limits statistical power, reduces the reliability of gender comparisons, and restricts the generalizability of the findings to a broader population of professors. In addition, the analysis relies primarily on non-parametric tests due to the small sample size and the non-normal distribution of several variables, which may limit the ability to detect subtle differences. Several variables analysed (e.g., religion, birthplace, parental occupation) are only loosely related to the main research question but could act as potential confounders. Due to limitations in sample size and data availability, we were unable to control for these factors, which should be considered when interpreting the results. Moreover, the professors’ biographies cannot be controlled for completeness and often information on their career paths before joining the University of Rostock is limited. Therefore, variables like median age at appointment do not necessarily reflect the age at first appointment as a full professor. Further, the division of epochs in this analysis follows the categorization already established in the catalogue, which we adopted without modification. Defining historical periods in fixed intervals can be challenging, as individual events often overlap and transitions between epochs may be gradual rather than discrete. Moreover, academic structures changed substantially over the centuries, which may affect comparability across epochs. Consequently, this approach may oversimplify complex historical developments and obscure nuanced temporal patterns in the data. Lastly, this study employs a binary classification of sex, thereby excluding non-binary and other gender identities. This approach introduces a potential bias, particularly when interpreting the proportion of women among professors in medicine. By relying on historical records that classify individuals solely as male or female, the data may over- or underestimate the representation of women and obscure the complexities of gender identity, especially in earlier periods when gender diversity was rarely documented or recognized.

### Future research

Future research should investigate temporal trends at other universities in Germany and internationally to assess regional differences. Given the historical division of Germany into East and West, and the persisting economic, societal, and political differences between the formerly separated regions, it would also be valuable to compare our results with those from a university in the western federal states. Comparative analyses with other faculties at the University of Rostock would be valuable as well. Furthermore, adopting a more inclusive classification of sex and gender could provide deeper insights into the representation of women and other underrepresented groups in academia. Given that a notable share of professors took up positions at other universities following their tenure at the University of Rostock, future research could explore the prevalence of such mobility across other medical faculties and academic disciplines. It would also be informative to determine whether certain universities are disproportionately favoured within particular fields. Further research is needed to better understand the structural and social factors underlying the increasing feminization of medicine and dentistry, as well as the contrasting patterns observed in other fields such as engineering and STEM. In particular, it would be important to investigate institutional policies, career trajectories after PhD completion, and the role of family formation and parenthood in academic career progression. Understanding why some graduates remain in academia while others transition to private practice could help universities design policies that support gender equality and prevent the loss of talented female academics.

## Conclusions

While women are overrepresented in the lower ranks of academia, they remain underrepresented in the highest ranks: In 2018, 63.16% of students, 59.69% of the faculty’s graduates, 59.50% of newly graduated doctors and 33.33% of habilitations were women, while only 10.94% of professors at the UMR were female. In comparison, in 2018 female professors accounted for 23.69% of all professors in medicine in Germany. The first initiative for the promotion of women at the UMR mentioned in the archive files dates to 1962 and 1964 the first female full professors joined the faculty. Over the course of the study period, the proportion of female professors at the medical faculty increased noticeably, which is also reflected in a significant difference in appointment years between male and female professors (*p* < 0.001). In 1985, 9.76% of professors were women, while 55.90% of students were female. By 2024, these figures had risen to 18.31% of professors and 69.38% of students, respectively. Although significant progress has been made, further systematic changes are required to achieve gender equity at the University of Rostock Medical Faculty. This will necessitate committed leadership, adequate resources, and continuous evaluation of implemented measures. Future research should compare trends with other national and international institutions, as well as with other faculties at the University of Rostock, to identify effective strategies. In addition, more conclusive studies on the impact of specific initiatives aimed at advancing women in academia are needed to inform the refinement of existing programs. Finally, it is important to consider the representation of individuals who do not fit into a binary sex system, in order to gain a more comprehensive understanding of equity and inclusion in academic settings.

## Data Availability

The data that support the findings of this study are available from the University of Rostock, but restrictions apply to the availability of these data, which were used under license for the current study, and so are not publicly available. Data are however available from the authors upon reasonable request and with permission of the University of Rostock.

## Appendix

### The historical context

The University of Rostock was found on the 12 November 1419 with papal approval. In the context of various disturbances within the city of Rostock, the university relocated its seat multiple times temporarily, first to Greifswald (1437–1443), then to Wismar and Lübeck (1487–1488). Following the university's conversion to Protestantism in 1531 as part of the Reformation, a challenging reconstruction phase ensued. This inevitably necessitated a clarification of the university's relationship with both the territorial lords and the city. In 1563, an agreement known as the *Formulae Concordiae* was reached. The territorial lords assumed patronage over the university, while the city council was granted co-patronage. As a result, two professorial colleges, the ducal (herzoglich) and the municipal (rätlich), were created and remained united by the council, with each patron assuming financial responsibility for their respective college. [69]

Around 1600, the university experienced its golden age thanks to the clear directives in the Formula Concordiae regarding the financing and organization of the university. Until around the mid-19th century, there were simultaneously ducal and municipal professors due to the co-patronage. [6]

In the mid-18th century, Duke Friedrich the Pious decided to reform the Theological Faculty of the university in accordance with Pietism. In response to the faculty's resistance to this decision, the duke obtained an imperial patent for the establishment of a new university. Therefore, a new university was to be opened in Bützow, while the university in Rostock faced closure. The ducal professors were thus ordered to Bützow, while the municipal professors remained in Rostock. However, during the Seven Years' War (1756–1763), Prussian troops arrived in Rostock, nullifying the duke’s authority, and the closure of the university in Rostock was never carried out. Consequently, from 1760 onwards, two universities coexisted in parallel in Mecklenburg. When the Duke died in 1785, the university in Bützow lost its primary supporter. His successor sought reconciliation with Rostock, leading to the reunification of the two universities in 1789, allowing them to resume joint academic operations. [70]

In 1827, the city relinquished its co-patronage in favour of Grand Duke Friedrich Franz I. Thanks to the financial support, particularly from Grand Duke Friedrich Franz II, the university flourished once again in the second half of the 19th century. Even during World War I, academic operations were maintained, and professors had to assume the additional duties of lecturers serving in the war. When Grand Duke Friedrich Franz IV abdicated in 1918, the Mecklenburg state government took over responsibility for the University of Rostock. During the Weimar Republic (1918–1933), the university worked on a new constitution that came into effect in 1932 but was abolished again a year later. [6]

Following the rise to power of the National Socialists in 1933, the university's self-governance was gradually restricted. The "Führerprinzip" which transferred the existing authorities of the council and senate to the rector, was implemented. From 1935 onwards, the rector was appointed by the Reich Ministry and was directly subordinate to it. [71]

After the end of World War II in 1945, the university underwent thorough denazification and reopened in February 1946. A fundamental restructuring occurred as part of the higher education reforms in Eastern Germany, with sections replacing faculties. [6]

In 1989, the political turnaround marked the end of the German Democratic Republic (GDR). Consequently, the faculty structure was reintroduced, and the university adopted the organizational structures of the Federal Republic of Germany. [6]

**Table 7:** Graduate and Doctoral Degree Statistics at UMR

Year (Source)	Total number of graduates UMR	Female graduates UMR	Total number of doctorates UMR	Female doctorates UMR
2024 [31]	418	266 (63.64%)	115	57 (49.57%)
2023 [31]	449	276 (61.47%)	137	80 (58.39%)
2022 [31]	402	255 (63.43%)	153	89 (58.17%)
2021 [31]	456	257 (56.36%)	162	84 (51.85%)
2020 [31]	393	236 (60.05%)	112	67 (59.82%)
2019 [31]	408	245 (60.05%)	112	65 (58.04%)
2018 [31]	392	234 (59.69%)	121	72 (59.50%)
2017 [17]	257	161 (62.65%)	126	69 (55%)
2016 [17]	276	178 (64.49%)	109	70 (64%)
2015 [17]	275	181 (65.82%)	124	77 (62%)
2014 [16]	273	162 (59.34%)	117	72 (61.53%)
2013 [16]	257	164 (63.81%)	118	76 (64.40%)
2012 [16]	281	192 (68.33%)	111	69 (62.16%)
2011 [16]	246	166 (67.48%)	92	49 (53.26%)
2010 [16]	252	187 (74.21%)	88	50 (56.81%)
WS 1998/99 [32]	139	67 (48.20%)	N/A	N/A
WS 1997/98 [32]	141	57 (40.43%)	N/A	N/A
WS 1996/97 [32]	85	43 (50.59%)	N/A	N/A
1985 [34]	154	95 (61.89%)	N/A	N/A

The year indicated varies depending on the data source. If only the year is given, it refers to both the winter semester (WS) and the summer semester (SS) with final examinations in that year, e.g., 2024 includes WS 2023/24 and SS 2024. The years 2010—2017 only include the degree programs in Human Medicine, Dentistry, and Medical Biotechnology. Source: University Statistics [31] & University of Rostock Archive Data

**Table 8:** Proportion of Female habilitations and professors at UMR

Year (Source)	Proportion of female habilitations UMR (%)	Proportion of female professors UMR (%)
2024	0.00	18.31
2023	0.00	16.92
2022	33.33	15.15
2021	47.37	13.85
2020	27.27	12.31
2019	50.00	10.45
2018	33.33	10.94
2017	20.00	6.56
2016	20.00	8.33
2015	25.00	8.47
2014	21.43	7.41
2013	16.67	9.09
2012	28.57	8.77
2011	0.00	8.77
2010	30.00	8.62
2009	33.33	7.14
2008	30.00	7.27
2007	10.00	10.17
2006	12.50	10.34
2005	0.00	10.71
2004	N/A	11.67
2003	N/A	0.00
2002	N/A	7.55
2001	N/A	5.56
2000	N/A	6.78
1985 [34]	N/A	9.76

Up to and including 2017, the figures refer exclusively to positions financed from the University of Rostock's budgetary funds; from 2018 onwards, third-party and special funds are also included in the calculation. However, these constitute only a very small proportion. Source: University Statistics, University of Rostock (not available to the public)

**Table 9:** Proportion of female professors in human medicine/health sciences in Germany

Year	Total number of professors in Medicine Germany	Number of female professors in Medicine Germany	Proportion of female professors in Medicine Germany (%)
2023	5229	1508	28.84
2022	5084	1445	28.42
2021	4920	1327	26.97
2020	4695	1208	25.73
2019	4442	1098	24.72
2018	4318	1023	23.69
2017	4158	924	22.22
2016	4042	864	21.38
2015	3848	791	20.56
2014	3789	726	19.16

Source: [30]

**Table 10:** Student Numbers at UMR

Year (Source)	Total number of students UMR	Number of female students UMR	Proportion of female students UMR
WS 2024/25	2649	1838	69.38
WS 2023/24	2591	1785	68.89
WS 2022/23	2535	1740	68.64
WS 2021/22	2448	1666	68.06
WS 2020/21	2431	1603	65.94
WS 2019/20	2340	1505	64.32
WS 2018/19	2370	1497	63.16
WS 2017/18 [17]	2347	1448	61.70
WS 2016/17 [17]	2296	1431	62.33
WS 2015/16 [17]	2268	1394	61.46
WS 2014/15 [16]	2216	1361	61.42
WS 2013/14 [16]	2149	1325	61.66
WS 2012/13 [16]	2134	1332	62.42
WS 2011/12 [16]	2087	1327	63.58
WS 2010/11 [16]	2035	1312	64.47
1985 [34]	1009	564	55.90
1974 [33]	890	N/A	73
1973 [33]	892	N/A	73
1972 [33]	851	N/A	74
1971 [33]	814	N/A	73

The year indicated varies depending on the data source. If only the year is given, it refers to both the winter semester (WS) and the summer semester (SS) with final examinations in that year, e.g., 2024 includes WS 2023/24 and SS 2024. The WS 2010/11—2017/18 only include the degree programs in Human Medicine, Dentistry, and Medical Biotechnology. Source: University Statistics & Archive Data, University of Rostock (not available to the public)
